# Research of Eliminating the Day-Boundary Discontinuities in GNSS Carrier Phase Time Transfer through Network Processing

**DOI:** 10.3390/s20092622

**Published:** 2020-05-04

**Authors:** Xiangbo Zhang, Ji Guo, Yonghui Hu, Dangli Zhao, Zaimin He

**Affiliations:** 1 University of Chinese Academy of Sciences, Beijing 100039, China; guoji@ntsc.ac.cn (J.G.); huyh@ntsc.ac.cn (Y.H.); zdli@ntsc.ac.cn (D.Z.); hezm@ntsc.ac.cn (Z.H.); 2 National Time Service Center, Chinese Academy of Sciences, Xi’an 710600, China; 3 Key Lab of Time-frequency Standard of Chinese Academy of Sciences, Xi’an 710600, China

**Keywords:** GNSS, precise point positioning, time and frequency transfer, day-boundary discontinuities, network, MGEX, frequency stability

## Abstract

Time and frequency transfer through global navigation satellite system (GNSS) precise point positioning (PPP) based on carrier-phase measurements has been widely used for clock comparisons in national timing laboratories. However, the time jumps up to one nanosecond at the day boundary epochs of adjacent daily batches lead to discontinuities in the time transfer results. Therefore, it is a major obstacle to achieve continuous carrier phase time transfer. The day-boundary discontinuities have been studied for many years, and they are believed to be caused by the long-term pseudorange noise during estimation of the clock offset in the daily batches and are nearly in accordance with a Gaussian curve. Several methods of eliminating the day-boundary discontinuity were proposed during the past fifteen years, such as shift and overlapping, longer batch processing, clock handover, and ambiguity stacking. Some errors and new noise limit the use of such methods in the long-term clock stability comparison. One of the effective methods is phase ambiguity fixing resolution in zero-differenced PPP, which is based on the precise products of wide-lane satellite bias (WSB) provided by the new international GNSS Service (IGS) Analysis Center of Centre National d’Etudes Spatiales (CNES) and Collecte Localisation Satellites (CLS). However, it is not suitable for new GNSS, such as the Beidou Satellite System (BDS), GALILEO, and QZSS. For overcoming the drawbacks above, Multi-GNSS Experiment (MGEX) observation data of 10 whole days from MJD 58624 to 58633have been network processed by batch least square resolution. These observations come from several ground receivers located in different national timing laboratories. Code and carrier phase ionosphere-free measurements of GPS and BDS satellites are used, and the time transfer results from network processing are compared with PPP results provided by Bureau International des Poids et Mesures (BIPM) and used for international atomic time (TAI) computation (TAIPPP) and universal time coordination (UTC). It is shown that the time offsets of three different time links are almost continuous and the day-boundary discontinuities are sharply eliminated by network processing, although a little extent of day-boundary discontinuities still exist in the results of UTC(USNO)-UTC(PTB). The accuracy of time transfer has been significantly improved, and the frequency stability of UTC(NTSC)-UTC(PTB) can be up to 6.8 × 10^−15^ on average time of more than one day. Thus, it is suitable for continuous multi-GNSS time transfer, especially for long-term clock stability comparison.

## 1. Introduction

GNSS are widely used in many national timing laboratories for time and frequency transfer or clock comparison [1,2,3,4,5,6,7,8,9] because of the high accuracy level and more economical from individual stations. Time transfer through GPSPPP, based on code and carrier-phase measurements, is one of the most commonly used methods. Since 2009, GPS PPP has been recommended for international time comparison by BIPM, and its high precision is widely recognized for about 100 picoseconds at each epoch [10,11], so more than 30 timing institutions have established such time links. However, the major problem of the day-boundary discontinuities that show time jumps in the boundary epochs of two adjacent batches can reach even more than one nanosecond, which limit PPP used for continuous precise time transfer. Many researchers have studied the problem of the day-boundary discontinuity for more than 15 years and believe that these discontinuities are in fact caused by the measurement noise of code-pseudorange during estimation of the station clock offset in 24 h batches because the station clock obtained is reliant on the code measurements [12,13,14]. The code-pseudorange noise is sometimes and for some stations not white noise, due to near-field multipath effects or variation of instrumental delays. The averaging of this colored pseudorange noise induces clock datum changes between daily batches at the level of a few hundred picoseconds to a few nanoseconds. IGS uses daily batches of GPS observations to estimate the precise satellite orbit and clocks. Therefore, there are boundary jumps visible from day to day in the products. PPP is based on the IGS estimates and therefore may inherit this effect [15]. Some researchers also believe day-boundary discontinuities in PPP results are caused by the inconsistency of phase ambiguity between two independent days [16,17]. In order to reduce the effect of the day-boundary discontinuities to PPP for continuous time transfer, the easiest way is to concatenate the daily solutions by using overlapping computation batches [18,19]. However, there is a drawback of this approach because it induces an undetected error in a single batch solution on the entire continuous time series. To overcome this problem, increasing the length of one batch from 24 h to longer batches, for example over three days, was introduced and tested. It was found that this process can obtain the continuous time series in internal processing batches by increasing the length of the computing batches, but there are still discontinuities at the batch boundaries. Furthermore, the required computer resources unfortunately increase nonlinearly. Solutions including more than three days in one batch seem to be impractical in routine processing. One possibility to average longer batches without increasing the required computer power is clock handover and ambiguity stacking [20], which is to store the initial phase ambiguities in daily normal equation matrices, and then stack them together over several days. However, it is not practicable due to the large number of clock parameters in a stacked normal equation. 

A new method for integer ambiguity fixing on un-differenced phase measurements for dual-frequency GPS was introduced by Laurichesse in 2009 [21] and successfully applied to estimate the clocks for PPP of ground-based receivers. Test results of this method for time transfer is continuous and it proved that it is very suitable used for long-term clock stability comparison. However, this method is based on the products of wide-lane satellite bias (WSB) from CNES and is applied only for GPS and GLONASS, but not for new GNSS, such as BDS and GALILEO.

The main objective of this paper is to focus on taking advantage of continuous PPP solutions analyzed as Orgiazzi et al. [22] and attempt to eliminate the day-boundary discontinuities through network processing of batch least square resolution, using multiple days as one data batch. In Section 2, we introduce the conventional daily PPP principle and provide background information on BIPM implementation of the PPP algorithm for TAI. Meanwhile, the problem of day-boundary discontinuities in some important stations and its statistical characteristics is also introduced. In Section 3, we propose the method of network processing using five days as one batch and explain the reason of eliminating the day-boundary discontinuities. In Section 4, experiments were setup using MGEX data of GPS and BDS to evaluate the performance of the network solution. Section 5 the results of time transfer through PPP and network processing were analyzed and compared to TAIPPP results provided by BIPM. Finally, Section 5 presents the conclusions and future proposals.

## 2. Materials and Methods

### 2.1. Data Processing Principle

The basic observation equations for GPS code-pseudorange and carrier-phase measurements ground of a station r to a satellite S have the following forms, respectively:

(1) $$P_{i}=\rho +c(dt_{r}-dT^{s})+d_{orb}^{s}+d_{trop}+d_{ion/P_{i}}+c(\Delta b_{r}+\Delta B^{s})+d_{mul/P_{i}}+\varepsilon_{P_{i}}$$

(2) $$\Phi_{i}=\rho +c(dt_{r}-dT^{s})+d_{orb}^{s}+d_{trop}-d_{ion/\Phi_{i}}+\lambda_{i}N_{i}+c(\Delta b_{r}+\Delta B^{s})+\varepsilon_{\Phi_{i}}$$

where and $\Phi_{i}$ are the code-pseudorange and carrier phase measurements in metric units for GPS L1 and L2 respectively, ρ is geometric distance between station r and satellite S,$dt_{r}$ and $dT^{s}$ are the clock errors for receiver and satellite with respect to the GPS time respectively, $d_{ion}$ and $d_{trop}$ are the ionospheric delay and tropospheric delay respectively; $\varepsilon_{P_{i}}$ and $\varepsilon_{\Phi_{i}}$ are relevant system noise and un-modeled residual errors including the multipath corrections; $N_{i}$ is initial ambiguity parameter for phase measurements and $\lambda_{i}$ is the wavelength of GPS carrier frequencies. The tropospheric delay is expressed as the sum of the hydrostatic and wet delays, both being the product between a given mapping function (mf) and the zenith path delay (zpd). The hydrostatic part is always stable and can be introduced using a model with the dry Niell mapping function. The wet part changes quickly and can be introduced using the Niell wet mapping function. The wet zpd is estimated with a 2 h sampling rate, with linear interpolation between theepochs of consecutively estimated zpd.

The main difference between the code-pseudorange and carrier-phase measurements is that, in the case of the phase measurements, it has equal but opposite ionospheric delays compared to code-pseudorange. The phase ambiguities in phase measurements have to be estimated and they are usually constant per satellite pass for every station (as long as the receiver keeps phase locked), whereas clock parameters will be estimated every epoch. This means it is impossible to directly estimate the receiver clock if only the carrier phase measurements are used. The code-pseudorange observations have no ambiguity parameter, and it can direct estimate the clock parameters. So, it is possible to use both observations in a combined data analysis, where a low weight is given to the code-pseudorange and high weight is given to carrier phase measurements, because code-pseudorang measurement is much noisier than carrier phase. So, in order to reduce the code errors from the meter lever to typically cm level, the code-pseudorange measurements are usually smoothed using the phase with a hatch filter. The observations are used at the 30 s sampling rate. The satellite positions can be obtained after interpolation on 12 points to IGS sp3 files in which the satellite positions are given at a 15 min sampling rate. The satellite clock errors can be corrected using the precise clock product with a 5 min sampling interval, provided by IGS or other GNSS analysis centers.

Traditionally, we use the code-pseudorange (P1 and P2) and carrier phase(L1 and L2) dual-frequency ionosphere-free combinations for PPP time transfer, and the forms are as the following equations after the corrections of satellite clock and orbits errors:

(3) $$P_{IF}^{G}=\frac{f_{1}^{2}P_{1}^{G}-f_{2}^{2}P_{2}^{G}}{f_{1}^{2}-f_{2}^{2}}=\rho^{G}+(cdt_{r}^{G}+b_{P_{IF}}^{G})+d{+}_{trop}B_{P_{IF}}^{G}+d_{mul/P_{IF}}^{G}+\varepsilon_{P_{IF}}^{G}$$

(4) $$\Phi_{IF}^{G}=\frac{f_{1}^{2}\Phi_{1}^{G}-f_{2}^{2}\Phi_{2}^{G}}{f_{1}^{2}-f_{2}^{2}}=\rho^{G}+(cdt_{r}^{G}+b_{\Phi_{IF}}^{G})+d_{trop}B_{\Phi_{IF}}^{G}+\lambda_{IF}^{G}N_{IF}^{G}+d_{mul/\Phi_{IF}}^{G}+\varepsilon_{P_{IF}}^{G}$$

where $P_{IF}^{G}$ and $\Phi_{IF}^{G}$ are combined code-pseudorange and phase observations, $N_{IF}^{G}=\frac{cfN_{1}{}_{1}-cf_{2}N_{2}}{f_{1}^{2}-f_{2}^{2}}$ is the combined phase ambiguity, and $b_{P_{IF}}^{G}$, $B_{P_{IF}}^{G}$, $b_{\Phi_{IF}}^{G}$, and $B_{\Phi_{IF}}^{G}$ are receiver and satellite internal hardware delays, respectively.

After linearizing the Equations (3) and (4), the parameters including receiver’s precise position ( in static or kinematic mode), receiver clock differences, zenith tropospheric delays, and combined carrier phase ambiguities of each satellite can be estimated using a sequential least squares scheme, with a weighting for the codes and carrier phases associated with the noise level of each observation type and satellite elevation. The traditional model has the advantages of simplicity, simple calculation and fewer estimated parameters, so it is a commonly used model for GPS PPP. However, due to the effects of hardware delays in both code-pseudorange and phase measurements, the combined ambiguity parameter is a float term, loses integer characteristics. The accuracy of PPP solution can reach few centimeters in position and 100 ns for clock differences respectively. Because the station clock is referred to the IGS Time scale (IGST) derived from the satellite clocks in the IGS products, so the differences between different station clocks can be inferred from subsequent subtraction.

### 2.2. Multi-Day Batch Solutions in TAIPPP

The PPP processing by BIPM for TAI computation was performed through NRCan-PPP software developed by Geodetic Survey Division of Natural Resources Canada, using IGS final 15-min satellite orbit and 5 min satellite clock products [23]. It processes long batch rinex files that span multiple days up to a maximum of 30 instead of daily batches in order to remove the day discontinuities, and then removes high frequency noises with Vondrak–Cepek smoothing [9]. The PPP could generate continuous phase-connected clock solution through the multi-day batches so that the clock stability at averaging times of interest (hours to days) can be improved. In the multi-day PPP processing, IGS final SP3 orbits and 5 min clock products are input as daily files. 

### 2.3. Combined of the Multi-GNSS Observations

With rapid development of BDS established by China recently, many studies have been conducted in order to evaluate its time and frequency transfer performance [24,25]. It is also expected that combining of GPS, BDS and other GNSS, will provide improved accuracy for the remote comparison of atomic clocks and timescale. Since GPS, BDS, and GALILEO satellite signals use the same signal structure of code division multiple access (CDMA), the same method can be used to build a combined GPS+BDS PPP observation model. However, due to the different individual receiver reference time, receiver inter-system bias (ISB) need to be added to unify the GPS and BDS receiver clock difference. ISB depends not only on the specific receiver, but varies with the GNSS clock product that defines the system time scales for the individual constellations [26]. So, on the basis of defining ISB of reference station as zero, ISBs and receiver clock offsets of other stations can be estimated in network processing. Moreover, the satellite clock corrections obtained in a combined analysis of the GPS and BDS observations refer to one and the same reference clock in the network solution. Because GLONASS transmit signals with frequency division multiple access (FDMA), so it has different frequency for each satellite. This results in significant differences of the satellite-related code hardware delays in each receiving channel. Therefore, it is necessary to carefully consider the pseudorange observations When combining GLONASS in multi-GNSS PPP. So, using Equations (3) and (4) and considering GPS time as a reference time system, the un-differenced ionosphere-free linear combinations of GPS and BDS observations can be written as Equation (5):

(5) $$\left.\begin{array}{l} P_{IF}^{G}=\rho^{G}+cdt_{r}+d_{trop}+\varepsilon_{P_{IF}}^{G}\\ \Phi_{IF}^{G}=\rho^{G}+cdt_{r}+d_{trop}+N_{IF}^{G}+\varepsilon_{\Phi_{IF}}^{G}\\ P_{IF}^{C}=\rho^{C}+cdt_{r}+ISB_{r}^{GC}+d_{trop}+\varepsilon_{P_{IF}}^{C}\\ \Phi_{IF}^{C}=\rho^{C}+cdt_{r}+ISB_{r}^{GC}+d_{trop}+N_{IF}^{C}+\varepsilon_{\Phi_{IF}}^{C} \end{array}\right\}$$

where G and C refer to GPS and BDS observations respectively, and ISB is the inter-system bias. As can be seen from above equation, ambiguity parameter is also a non-integer term when receiver and satellite code biases are un-calibrated, so day-boundary discontinuities will still exist in the results of combined multi-GNSS PPP.

### 2.4. The Problem of the Day-boundary Discontinuities in Combined Multi-GNSS PPP

It is known that code-pseudorange measurements must be used in order to obtain the unbiased estimation of the receiver clock difference at the initial epoch. The measurement noise in code-pseudorange cannot be ignored and it leads to biased combined phase ambiguity. There is an uncertainty in the phase ambiguity calculated by each batch of data processing relative to the “true value” (unbiased estimation).Therefore, a time jumps come out at the day boundary epochs of two adjacent batches, that is “the day boundary discontinuity”. According to Yao and Defraigne [15,17], the noises that affect the results of PPP are colored noises, and it was found that there are many other original affects that contribute to the boundary discontinuity, such as the discontinuity of IGS products, multipath, imperfect modeling of the tropospheric mapping function, and precision of station position. It also may come from batch length data of PPP and the algorithm of fixing phase ambiguity. Moreover, the existence of the hardware delays originating in the receiver and satellite also result in the day-boundary discontinuity includes because the phase ambiguity is incorrect when using the algorithm to fix ambiguity in PPP.

**Figure 1:**
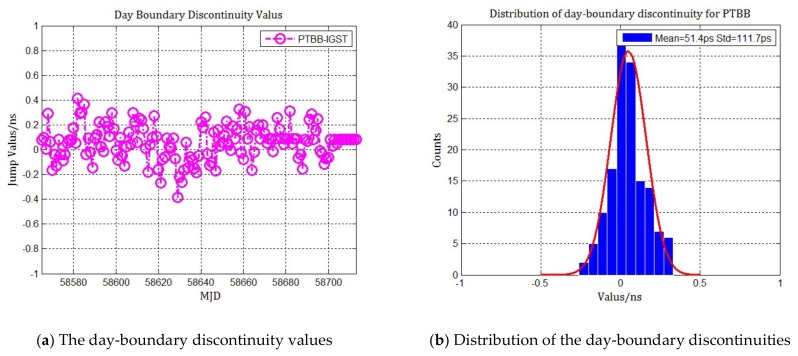
Statistics of the day boundary discontinuities and distribution in combined multi-GNSS PPP for PTBB. (**a**) The day-boundary discontinuity values in nanoseconds; (**b**) The distribution of the day-boundary discontinuities compared to Gaussian fitting.

**Figure 2:**
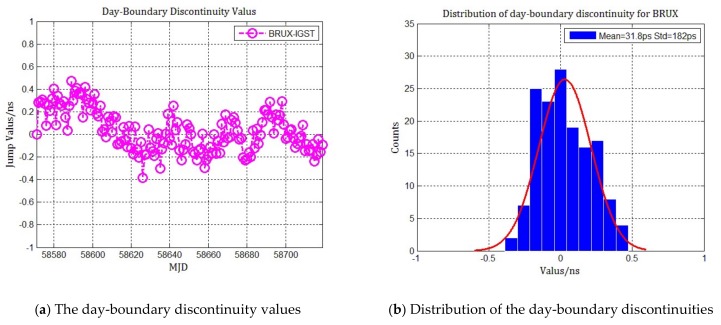
Statistics of the day-boundary discontinuities and distribution in combined multi-GNSS PPP for BRUX. (**a**) The day-boundary discontinuity values in nanoseconds; (**b**) The distribution of the day-boundary discontinuities compared to Gaussian fitting.

In order to analyze the statistical distribution of the day-boundary discontinuities in combined multi-GNSS PPP, the MGEX tracking stations of PTBB, BRUX with multi-GNSS observations were selected and their day-boundary discontinuities were counted and analyzed, as Figure 1 and Figure 2 show. The time span in the statistics results is 150 days from MJD 58570 to MJD 58720, and the precise satellite orbit and clock products are provided by Centre for Orbit Determination in Europe (CODE) with 5 min and 30 s sample interval rate respectively. It can be seen from the distribution that the average of day-boundary discontinuities in all receiver clock offsets is less than 100 picoseconds, and the standard deviation is less than 200 picoseconds. The probability density is nearly in accordance with the Gaussian distribution, indicating that the day-boundary discontinuities are approximately white noise. This result matches well with other authors’ studies of the day-boundary discontinuity statistical distribution in GPS PPP.

## 3. GNSS Carrier-Phase Time Transfer through Network Processing

As mentioned above, there are many factors that cause the day-boundary discontinuities when estimating the station clock offset in PPP, including the discontinuity of precise orbit and clock products. So, in order to overcome the impact of relying highly on the precise satellite products provided by IGS, a method of batch least square network processing is proposed. Generally, there are several steps in the network processing and the first is use a station with good stability of an external reference time as a fixed reference station in the selected stations. Secondly, the data are processed in a similar non-differential principle like PPP to obtain clock differences of satellites and all stations relative to reference station, with the dual-frequency iono-free measurements of GPS and BDS. Finally, the relative clock differences between two stations should be single differenced epoch by epoch so that the time transfer results can be obtained. Through this processing, the connection can be established between different station observation equations, and the time transfer results can be obtained using relative clock difference when one reference clock is fixed.

When the station ref is input as the reference station, the clock offset $dT^{S}$ of satellite S is relative to the reference clock $dt_{ref}$ is $d{\bar{T}}^{S}$, as Equation (6) shows:

(6) $$d{\bar{T}}^{S}=dT^{S}-dt_{ref}$$

Similarly, the receiver clock offset of any station k relative to the fixed reference station is shown in Equation (7):

(7) $$d{\bar{t}}_{k}=dt_{k}-dt_{ref}$$

After the reference station clock is selected and fixed, the observation equations of station k no longer includes the parameters of the station receiver clock offsets, but clock offsets relative to the reference station clock, tropospheric delay and phase ambiguity parameters instead. The observation equations of station k relative to the fixed reference station are according to the forms of Equations (8) and (9):

(8) $$\nu_{k,P}^{S}=d{\bar{t}}_{k}-d{\bar{T}}^{S}+d_{k,trop}/c+\rho_{k}^{S}/c+\varepsilon_{k,P}^{S}-P_{k}^{S}/c$$

(9) $$\nu_{k,\Phi }^{S}=d{\bar{t}}_{k}-d{\bar{T}}^{S}+d_{k,Trop}/c+\lambda N_{k}^{S}/c+\rho_{k}^{S}/c+\varepsilon_{k,\Phi }^{S}-\lambda \Phi_{k}^{S}/c$$

where $d{\bar{t}}_{k}$ is the time transfer result between station k and station ref. After single difference, the clock differences between different stations can be inferred.

It can be seen that the observation equations of the network processing has the exactly same form as the traditional PPP (based on the least-squares algorithm that minimizes measurement residuals solving for orbits, satellite and station clock offsets, non-integer phase ambiguities and zenith tropospheric delays), except that the satellite and receiver clock offsets change into the clock offsets relative to the fixed reference station. Compared to the daily PPP, the network method does not require the precise satellite clock products provided by IGS, but all clock offsets of satellites and stations are estimated with respect to the chosen reference clock. The precise coordinates of stations from PPP can be used as input values, and the satellite orbit is based on the orbit predicted by IGS MGEX. Unlike PPP, a single-station solution although several stations can be processed together for convenience, the estimates of each station by the network solution can benefit from the measurements of all stations being in principle more robust and precise [27]. It is believed that GNSS network solution can achieve the same precision as the traditional post-processed PPP but the uncertainty of ambiguities between batches is lower than PPP because it can avoid the discontinuity of satellite precise products used and the hardware delays can be canceled out during the process of differenced. Therefore, it can remove the time jumps in the batch boundary that are induced by the uncertainty of ambiguities. If the idea of multi-day batch is adopted again, most of the day-boundary discontinuities in the time transfer should be eliminated, and continuous clock time series can be obtained. It can be implemented for global GNSS network of timing laboratories.

## 4. Experiment Setup

In order to verify the effect of the network processing on eliminating the day-boundary discontinuities and it is suitable for continuous time transfer, several ground MGEX stations with GPS and BDS observations were selected to form the network. These stations are operated by national timing laboratories participating in UTC/TAI computation and receivers are connected to external H-masers. Un-differenced dual frequency iono-free combination of GPS and BDS measurements are used as input data. The processing is based on the batch least square adjustment resolution, using five days as one data batch. Five of the selected stations and related equipment configuration are shown in Table 1. Considering that the German Federal Institute of Physics (PTB) is usually selected as the intermediate station in international time transfer, so PTBB is selected as the fixed reference station for network processing. At the same time, in order to analyze and evaluate effect of eliminating the day-boundary discontinuities for time transfer of carrier phase measurements through network method, three typical links of different lengths, SPT0-PTBB (620 km), USN8-PTBB (6270 km) and NTP3-PTBB (7170 km), are formed using the above stations. The total time length of data that are processed is 10 whole days from MJD 58624 to 58633. The time intervals of clock offsets for selected stations are 5 min in order to synchronize with TAIPPP results that BIPM provided. Meanwhile, multi-GNSS combined PPP with the daily batch was performed using the precise orbit and clock products provided by CODE. For the sake of a clear comparison, the results of TAIPPP are used as a reference and differences between TAIPPP and the daily PPP network processing are statistically analyzed.

## 5. Results and Discussion

### 5.1. European Continental Baseline (SPT0-PTB ~620 km)

SPT0 is not only an IGS station and but also a BIPM station used for TAI calculation, it is located in the Swedish National Institute of Technology and is driven by UTC(SP). PTBB is also an IGS station located in PTB (Braunschweig, Germany), it is directly driven by UTC(PTB).It is about 620 km between SPT0 and PTBB, so multi-GNSS PPP and network processing are respectively used to calculate the clock offsets of such medium time link in Europe to compare UTC (SP) -UTC (PTB) with the results provided by BIPM. It is also used to evaluate the effectiveness of reducingthe level of day-boundary discontinuities and improvingthe robustnessand the frequency stability of time transfer base on carrier phase measurements.

**Figure 3:**
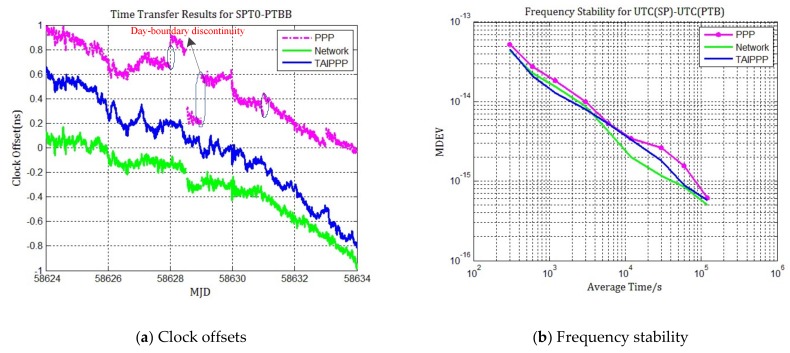
Time transfer and frequency stability results for UTC (SP)-UTC (PTB). (**a**) Station clock offsets of PPP (purple), network solution (green) and TAIPPP (blue), for MJD 58624–58633; (**b**)Frequency stability for PPP (purple), network solution (green) and TAIPPP (blue).

Figure 3a shows the differences of UTC(SP)-UTC(PTB) obtained by PPP, network solution and TAIPPP with a time interval of 5 min. Figure 3b shows the frequency stability of UTC(SP)-UTC(PTB) performed by modified Allan deviation (MDEV), which can distinguish white phase-modulation noise and flicker phase-modulation noise (slopes are -2/3 and -1, respectively) that characterize a frequency source noise model.

It can be clearly seen that, with reference to the results of TAIPPP, there are noticeable jumps in the time series of PPP at the boundary epochs of MJD 58627–58628, 58628–58629, 58630–58631, and 5,8632–5,8633, leading to discontinuous time transfer results. But there is no such discontinuity in network processing results and the time series is almost continuous. The slope of network processing results also matches the tendency of TAIPPP quite well. From MDEV of time transfer results obtained with daily PPP, network solution and TAIPPP (Figure 3b), it can be clear seen that the frequency stability of network solution is better than daily PPP results, especially at the average time of one day. It is slightly worse (<4000 s) for short-term frequency stability of the network solution than TAIPPP due to a higher short-term noise in network solution. However, when the average time is greater than 4000 s, the network solution once again becomes better.

The differences that daily PPP and network solution respected to TAIPPP are also calculated. The standard deviation of the difference between daily PPP and TAIPPP is 0.141 ns, but it is only 0.113 ns for the standard deviation of difference between network processing and TAIPPP, which indicates that the measurement noise of time link by network processing is lower and the time transfer accuracy is better compared to the daily PPP. There is a drift about 0.5 ns because the linear trend was not removed in order to clearly show the originally change of difference between two time scales.

The average absolute value of day-boundary discontinuities and their standard deviation areas well calculated and summarized in Table 2 for selected cases of the daily PPP and network solution. These day-boundary discontinuities are evaluated as the difference in between the final 5 min estimates of day i and the first 5 min estimates of day i+1.

**Figure 4:**
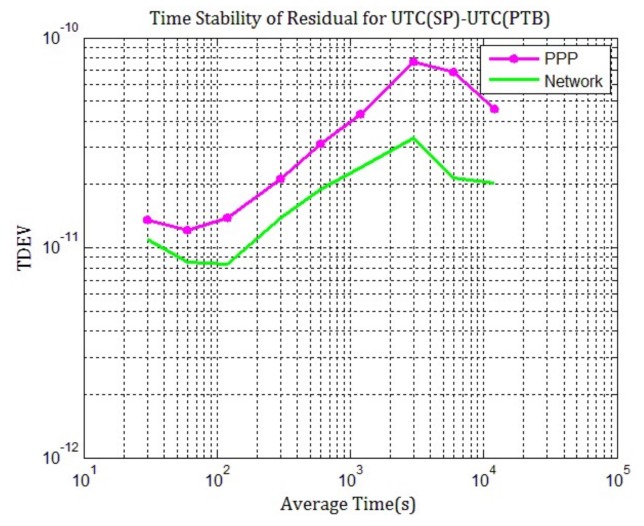
TDEV of the residuals between PPP (purple), network solution (green) and TAIPPP provided by BIPM from MJD 58624 to 58633.

Furthermore, the time deviation (TDEV) of residuals between PPP and network solution are compared in order to characterize how well PPP and network processing results match TAIPPP mathematically and how the time stability is better for the time link, which is shown in Figure 4. It can be clearly seen that “Network–TAIPPP” has the smallest fractional frequency in a long term (>10,000 s), which means that the network processing results match TAIPPP best and time stability is better. This once again confirms our observation in Figure 3 and shows that network solution can improve the accuracy and frequency stability of time transfer with carrier phase measurements.

### 5.2. Europe-North America Transatlantic Baseline (USN8-PTBB ~ 6270km)

**Figure 5:**
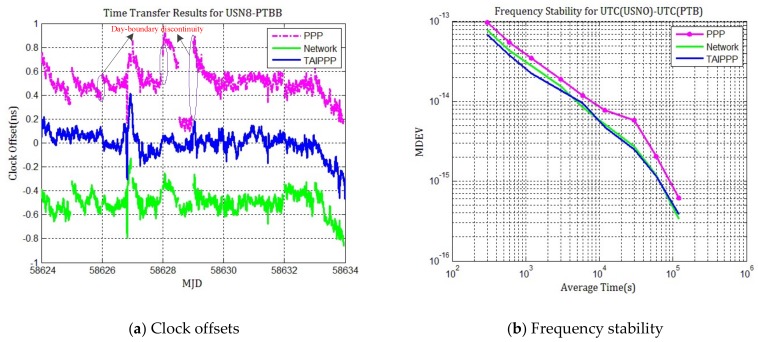
Time transfer and frequency stability results for UTC(USNO)-UTC(PTB). (**a**) Station clock offsets for 10 day batches using PPP (purple), network processing (green) and TAIPPP solutions (blue); (**b**)Frequency stability for PPP (purple), network processing (green) and TAIPPP solutions (blue).

USN8 is an IGS MGEX station located at the US Naval Observatory and the receiver on it is driven by UTC (USNO) with no corrections. The aim is to evaluate the time transfer between the USN8 and PTBB stations to compute UTC (USNO)-UTC (PTB) through network processing and compare results to the TAIPPP. There are 10 days of combined multi-GNSS observations that have been split into two batches of five days. The time transfer results are shown in Figure 5.

From Figure 5a, it can be seen that although the network processing results still have “time jumps” between boundaries of MJD 58625, 58627, and 58632, its magnitude decreases a lot and the trend of the clock difference curve agrees TAIPPP very well. It is significantly reduced compared to daily PPP results, which exactly shows that the day-boundary discontinuities can be eliminated through network solution. What needs to be explained here is that there are many other causes for the remaining discontinuities of network processing results in the link of USN8-PTBB, like precision of the station position of USN8 and multipath errors. It needs further research in the later work. In addition, from the MDEV results of PPP, network processing, and TAIPPP, it can be seen that the frequency stability of the network processing is better than PPP, especially when the average time is more than one day. Therefore, it is better approximated to the long-term frequency stability of TAIPPP.

Similarly, we also calculate the average absolute value of day-boundary discontinuities and their standard deviation for selected cases of daily PPP and multiple-day network solution, as Table 3 summarized.

**Figure 6:**
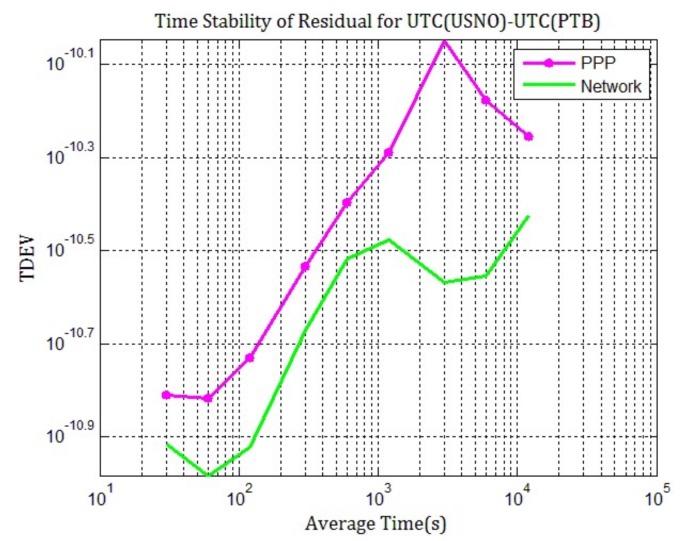
TDEV of the double-difference for TAIPP with respect to PPP (purple), network solution (green), from MJD 58624 to 58633.

Meanwhile, double-differences of network solution and PPP respect to TAIPPP are calculated. The standard deviation of the difference between TAIPPP and network solution is only 0.078 ns, but it is 0.12 for the difference between TAIPPP and the daily PPP. The same conclusions can be drawn as for Figure 4. The measurement noise of time transfer by network solution is lower and the accuracy is better than PPP. From the TDEV of the differences between daily PPP and network solution compared to TAIPPP, as Figure 6 shows, it can be seen that it has a smaller fractional frequency in a long term (>10,000 s) for network solution results than for PPP. This once again confirms our observation according to Figure 5.

### 5.3. European-Asia Ultra-Long Baseline (NTP3-PTBB ~ 7170 km)

**Figure 7:**
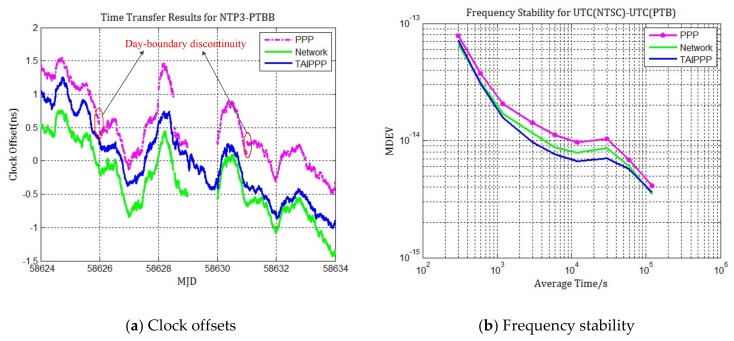
Time transfer and frequency stability results for UTC(NTSC)-UTC(PTB). (**a**) Station clock offsets for 10 day batches using PPP (purple), network processing (green) and TAIPPP solutions (blue); (**b**)Frequency stability for PPP (purple), network processing (green) and TAIPPP solutions (blue).

As an example of the ultra-length baseline between Europe and Asia, the pair of stations NTP3 and PTBB apart nearly 7200 km is chosen for evaluating the performance of time transfer through network solution. NTP3 is a station located at the National Time Service Center of Chinese Academy of Sciences and the receiver on it is driven by UTC (NTSC). Since there are no observation data on MJD 58629 for the station of NTP3, the time span for comparison between NTP3 and PTBB is only nine days. Figure 7 presents the difference of UTC(NTSC)-UTC(PTB) and MDEV of the three techniques, PPP (purple), network solution (green) and BIPM TAIPPP (blue circles). It is clearly noticeable that there is a better agreement between network solution and TAIPPP than between PPP and TAIPPP.

From Figure 7a, the time series of the multi-day network solution is continuous, except that there is no result in MJD 58629. The slope is also very consistent with TAIPPP result. However, it is observed from the result of daily PPP that there is obvious bias about 0.3 ns at the boundary epochs of MJD 58626 and 58631. In addition, it can be seen from the MDEV of three techniques that the frequency stability of network solution is almost same as the frequency stability of TAIPPP, although a small higher short-term noise still exists in the network solution, and it is better than the PPP result. When the average time is greater than one day, the frequency stability can reach 6.8 × 10^−15^, which indicates that it can truly reflect the relative change of UTC(NTSC) -UTC(PTB) through network solution rather than PPP.

The average absolute value of day-boundary discontinuities and their standard deviation are summarized in Table 4.

**Figure 8:**
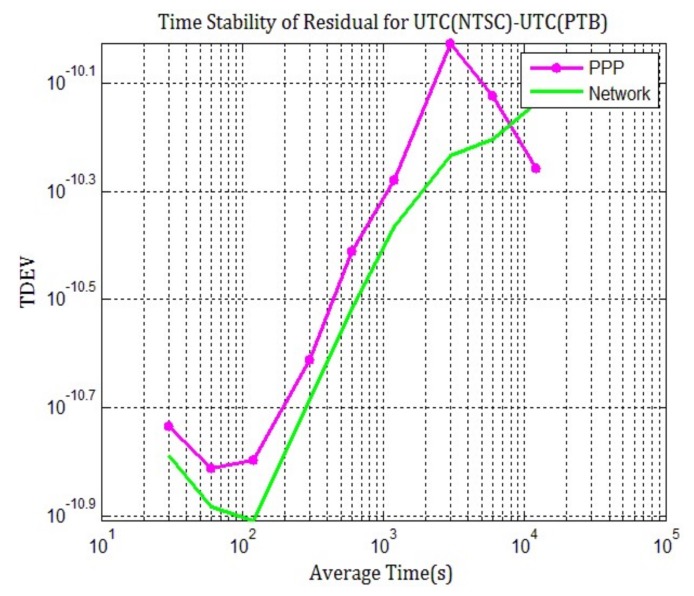
TDEV results for the double-difference of TAIPP with respect to daily PPP (purple), and network solution (green), from MJD 58624 to 58633.

The differences between daily PPP and network solution compared to TAIPPP are calculated, and the standard deviations of the difference between TAIPPP and the two GNSS techniques are 0.17 ns and 0.14 ns respectively, indicating that measurement noise of network solution once again smaller than PPP for such ultra-long time link. From the TDEV of the double-differences in Figure 8, it can be seen that, similar to other links, the frequency and time stability of GNSS carrier phase time transfer for ultra-long baseline can be improved through multi-day network solution than the daily PPP. Additionally, network solution offers almost the same tendency as TAIPPP, and it is more suitable for continuous multi-GNSS time and frequency transfer.

## 6. Conclusions and Future Prospects

With the aim of continuous GNSS carrier-phase time transfer, in this paper, the method of eliminating day-boundary discontinuity through network processing by batch least square resolution is proposed. A comparison analysis of the day-boundary discontinuities in time transfer results with three different baselines are presented, which were obtained with the traditional daily PPP, network processing, and TAIPPP provided by BIPM. The standard deviation of residuals and MDEV were used as the indicator for the improvement of time transfer accuracy and frequency stability for network processing. Furthermore, TDEV of double-differences between TAIPPP and the two GNSS techniques are also calculated and compared.

Three conclusions can be obtained from this study. Firstly, it can largely eliminate the level of day-boundary discontinuities through network processing with multiple days as one data batch for combined measurements of GPS and BDS. However, there are still some day-boundary discontinuities left in network processing results of USN8-PTBB due to unknown causes. Secondly, compared to TAIPPP, the standard deviation of difference between network processing and TAIPPP is sharply decreased compared to the standard deviation of difference between traditional daily PPP and TAIPPP, which indicates the impact of noise on time transfer link is largely reduced, and the time transfer accuracy is significantly improved it is more robust for the time links as well. Finally, MDEV of network solution is almost same as that of TAIPPP except from the slightly worse of short-term frequency stability due to the occurrence of a higher short-term noise, both frequency and time stability are better than the traditional daily PPP, especially when the average time is more than one day. Itis more suitable for continuous multi-GNSS time transfer through network solution, and it can truly reflect the changes of the time offset in two different timing laboratories and the stability of clocks.

Although it is more robust and precise when estimating the station clock and other parameters through network processing, the phase ambiguity is still float. Integer ambiguity fixing is a well-developed method in the precise time transfer of carrier phase measurements, and it has been successfully adapted for post-processed least-square analysis of daily GPS PPP and single-difference baseline. Therefore, our future research will be focus on network processing with integer carrier-phase ambiguity resolution using multi-GNSS observations. Additionally, the impact of discontinuities in MGEX products and other reasons that contribute to the boundary discontinuities in daily and longer batches will be also studied.

## Figures and Tables

**Table 1:** Geodetic Stations and Associated Equipment forSelected Timing Laboratories.

Station	Receiver	Country	Antenna	External Reference
PTBB	ASHTECH Z-XII3T	Germany	ASH700936E	UTC (PTB)
BRUX	SEPT POLARX4TR	Belgium	JAVRINGANT_DM	UTC (ROB)
SPT0	JAVAD TRE_G3TH DELTA	Sweden	JNSCR_C146-22-1	UTC (SP)
USN8	SEPT POLARX4TR	America	TPSCR.G5	UTC (USNO)
NTP3	SEPT POLARX4TR	China	SEPCHOKE_MC	UTC (NTSC)

**Table 2:** Average absolute value and standard deviation of the day boundary discontinuities for PPP and network solution.

Table	Daily PPP	Network Solution	TAIPPP
SPT0-PTBB	84.8 ± 125.5 ps	12.7 ± 58.4 ps	2.3 ± 8.1 ps

**Table 3:** Statistics of the day boundary discontinuities for PPP and Network solution.

Time Link	Daily PPP	Network Solution	TAIPPP
USN8-PTBB	167.5 ± 221.7 ps	12.5 ± 44.1 ps	2.7 ± 20.7 ps

**Table 4:** Statistics of the day boundary discontinuities for PPP and Network solution.

Time Link	Daily PPP	Network Solution	TAIPPP
NTP3-PTBB	69.1 ± 116.9 ps	12.7 ± 29.6 ps	4.1 ± 20.5 ps
